# *Xenopus* Oocyte As a Model System to Study Store-Operated Ca^2+^ Entry (SOCE)

**DOI:** 10.3389/fcell.2016.00066

**Published:** 2016-06-24

**Authors:** Raphaël Courjaret, Khaled Machaca

**Affiliations:** Department of Physiology and Biophysics, Weill Cornell Medicine Qatar, Education City, Qatar FoundationDoha, Qatar

**Keywords:** *Xenopus* oocyte, store-operated Ca^2+^ entry (SOCE), meiosis, confocal imaging, two-electrode voltage clamp, RNA injection

## Abstract

Store-operated Ca^2+^ entry (SOCE) is a ubiquitous Ca^2+^ influx pathway at the cell membrane that is regulated by Ca^2+^ content in intracellular stores. SOCE is important for a multitude of physiological processes, including muscle development, T-cell activation, and fertilization. Therefore, understanding the molecular regulation of SOCE is imperative. SOCE activation requires conformational and spatial changes in proteins located in both the endoplasmic reticulum and plasma membrane. This leads to the generation of an ionic current of very small amplitude. Both biochemical and electrophysiological parameters of SOCE can be difficult to record in small mammalian cells. In this protocol we present the different methodologies that enable the study of SOCE in a unique model system, the frog oocyte, which provides several advantages and have contributed significantly to our understanding of SOCE regulation.

## Introduction

Ca^2+^ is a ubiquitous intracellular messenger that activates various signal transduction cascades allowing cells to adapt to various queues in their environment. Cytoplasmic Ca^2+^ levels are maintained at low levels (on the order of 100 nM) providing a low baseline for Ca^2+^ transients, and as such enhancing signaling efficiency. In contrast to the low cytoplasmic Ca^2+^ levels, Ca^2+^ concentrations in the extracellular space are high, 1–2 mM. The other source for Ca^2+^ is intracellular stores, primarily in the lumen of the endoplasmic reticulum (ER) with Ca^2+^ concentrations in the hundreds of micromolar range (Demaurex and Frieden, 2003). This builds a large driving force for Ca^2+^ and cytoplasmic Ca^2+^ transients can be generated by Ca^2+^ release from intracellular stores, Ca^2+^ influx from the extracellular space or a combination of both. This is followed by Ca^2+^ extrusion out of the cell and Ca^2+^ reuptake into the ER lumen to return cytoplasmic Ca^2+^ back to its baseline resting values in preparation for the next signaling transient. The recycling of Ca^2+^ in the ER does not allow to compensate for the released ions and consequently the cells developed a refilling mechanism that allow to import Ca^2+^ from the extracellular space into intracellular ER stores. This Ca^2+^ influx pathway termed “Store-Operated Ca^2+^ Entry” (SOCE) and its required effectors have been identified in some protozoa as well as vertebrates and invertebrates but have not yet been identified in plants (Collins and Meyer, 2011; Bonza et al., 2013). SOCE not only contributes to the homeostasis of ER stores but also plays critical roles in shaping Ca^2+^ transients (Parekh and Putney, 2005; Prakriya and Lewis, 2015).

Interestingly SOCE links functionally and physically the ER stores and the cell membrane. The concept that intracellular store Ca^2+^ levels can regulate Ca^2+^ entry from the extracellular space was first proposed by Jim Putney in 1986 as the capacitative Ca^2+^ entry model (Putney, 1986), a system that could bypass the low Ca^2+^ concentration in the cytosol to refill the stores. SOCE was later shown to be a primary Ca^2+^ influx pathway in non-excitable cells with some functionality in excitable cells as well (Parekh and Putney, 2005). For example SOCE is critical for T-cell activation in response to antigen stimulation and defects in SOCE lead to immunodeficiency in humans (Serafini et al., 1995; Feske, 2010). Multiple molecular partners are involved in the generation of SOCE, the two key players being the ER luminal Ca^2+^ sensor STIM and the Ca^2+^ channel Orai at the plasma membrane. The depletion of intracellular Ca^2+^ stores leads to the unfolding of STIM and its aggregation into the so called puncta or clusters in the cortical ER that is located in close proximity to the plasma membrane (10–20 nm) (Luik et al., 2006, 2008; Liou et al., 2007; Hogan, 2015; Prakriya and Lewis, 2015). The clustered STIM protein then recruits the Ca^2+^ channel Orai in the puncta and promotes its activation and Ca^2+^ influx into the cell (Vig et al., 2006; Fahrner et al., 2009; Park et al., 2009; Hogan, 2015; Prakriya and Lewis, 2015). This generates a very well-defined Ca^2+^ microdomain at the ER-PM junction (Hogan, 2015).

Here we detail the protocols involved in the use of *Xenopus* oocytes as a model system to study SOCE both physiologically during meiosis and following heterologous expression of proteins that regulate SOCE. SOCE is down-regulated during M-phase of the cell cycle in both mitosis and meiosis. The oocyte is arrested in prophase I of meiosis and progresses to the metaphase II stage during the process of oocyte maturation in preparation for fertilization (Arredouani et al., 2010) and M-phase regulation of SOCE is the only known physiological situation where SOCE is completely inactivated. Therefore, understanding the mechanisms regulating SOCE during M-phase offers a unique opportunity to understand SOCE regulation. SOCE activation requires precise control of the sub-cellular localization of multiple proteins, including STIM, Orai and the Sarco/Endoplasmic Reticulum Ca^2+^ ATPase (SERCA) that localize to distinct cellular compartments. Imaging the spatial organization of SOCE regulators in small mammalian cells is a tedious task given the complexity of the spatial distribution of organelles such as the ER. The large size of the *Xenopus laevis* oocyte, which allows for good spatial separation between the ER and the plasma membrane makes it an excellent model to study the distribution of the membrane proteins involved in SOCE. Another limiting factor in mammalian cells is the SOCE current density that can be as small as 1 pA/pF. Although the density of the endogenous SOCE current in the *Xenopus* oocyte is similar to what is observed in mammalian cells (≈0.5 nA/nF), the large oocyte surface area (200–250 nF) results in a significant SOCE current (~100 nA) (Yao and Tsien, 1997; Machaca and Haun, 2000). In addition, the *Xenopus* oocyte expresses Ca^2+^-activated Chloride Channels (CaCC) that can be used as an amplifying system to report Ca^2+^ influx through SOCE (Machaca and Hartzell, 1999). CaCC allow the recording of SOCE in naïve oocytes as well as in cells overexpressing various SOCE proteins. The *Xenopus* oocyte, through injection of exogenous RNA, a method now more than 40 years old (Gurdon et al., 1971), is one of the major expression system for membrane proteins such as ion channels (Cens and Charnet, 2007) as well as other potential partners or regulators of the SOCE process.

## Materials and equipment

### *xenopus laevis* oocyte preparation

1. Animals: hCG (human chorionic gonadotropin) injected wild *Xenopus laevis* female frogs^1^ are obtained from *Xenopus* Express (Le Bourg, France), the animals are quarantined after shipment for 4 weeks in static aquaria before being transferred to a recirculating water system (Aquaneering, San Diego, CA). They live on a 12/12 dark/light photoperiod and are fed twice a week.
2. Anesthetics: Tricaine (Ethyl 3-aminobenzoate methanesulfonate, Sigma) is dissolved at a concentration of 5 g/L in deionized water containing NaHCO_3_ (2 g/L).
3. Solutions: nominally Ca^2+^-free solution Ringer contains 96 mM NaCl, 2 mM KCl, 5 mM MgCl_2_-6H_2_O, 5 mM Hepes. The pH is adjusted to 7.6 with NaOH. The Ca^2+^ containing solution is made by adding 0.6 mM Ca^2+^ to the Ca^2+^ free media, both solutions are filtered sterilized.
4. Enzyme: Collagenase IA (Affymetrix), is dissolved on the day of the surgery in Ca^2+^-free solution (2 mg/ml) and placed in 50 ml conical tubes (10 ml per tube).
5. The oocytes are stored in 12 cm Petri dishes at 18°C.
6. Oocyte media: The oocytes are kept in 0.5x L15 media (Sigma, L4386) supplemented with 10 mM Hepes, penicillin (10 units/mL), and streptomycin (10 μg/mL) and gentamycin (100 μg/mL), pH is adjusted to 7.6 with NaOH and the solution is filter sterilized. The L15 solutions are stored at 4°C and used within a week.

### RNA synthesis

RNA synthesis is performed using the “mMessage mMachine T7 Kit” according to the manufacturer instructions (Life Technologies).

### RNA injection

1. Injection needle: Injection needles are pulled from glass capillaries (3.000.203.G/X, Drummond) using a P2000 puller (Sutter Instruments), the tip is then broken with tweezers to generate a tip diameter of ~20 μm^2^.
2. Micro-injection setup: A plastic grid (1.5 mm mesh opening) is glued (Spectra Mesh, Spectrum Laboratories, Rancho Domingues, CA) at the bottom of a 50 mm plastic dish to hold the oocytes in place while injecting. The injection needle is mounted on a micro-injector (Nanoject II, Drummond) attached to a three axis manipulator. The injection is performed under a stereomicroscope (SZ61, Olympus). Some oocytes will die after injection and some will not express consequently, for imaging and electrophysiology, 25–50 oocytes are injected allowing to record 5–10 individual cells.

### Electrophysiological recordings

1. Sharp microelectrodes: Intracellular microelectrodes are pulled from glass capillaries (GC-150TF10, Harvard Apparatus) using a P97 puller (Sutter instruments). They have a resistance of 1~2 MΩ when filled with 3M KCl.
2. Recording setup: The two electrodes are connected to a GeneClamp 500B amplifier controlled by pClamp 10.5 (Axon Instruments). The signals are digitized using a Digidata 1322 (Axon Instruments). The reference electrode consists of a chlorinated silver wire.
3. Perfusion: The recording chamber is a custom made half bowl (~1 cm in diameter) milled in a block of plexiglas. The cells are continuously superfused using a peristaltic pump (Minipuls, Gilson). A gravity perfusion device is added to the system to allow fast application of drugs contained in 50 ml syringes. Both perfusion systems are connected to the recording chamber using a 4-way manifold to minimize the dead volume (MP Series, Warner Instruments).
4. Recording solutions: The composition of the standard Ringer solution is: 96 mM NaCl, 2.5 mM KCl, 1.8 mM CaCl_2_, 2 mM MgCl_2_, and 10 mM Hepes, pH adjusted to 7.4 with NaOH.

### Confocal imaging

1. Expression screening: The cells expressing fluorescent proteins are tested for expression using a stereomicroscope (Lumar V.12, Zeiss).
2. The oocytes are dropped in a holder made of a metal plate (0.8 mm thick) drilled with a hole of 1.3 mm in diameter. The grid is placed in a recording chamber whose bottom is a glass coverslip (Warner Instruments Series 20).
3. Confocal imaging: We use two different inverted confocal microscopes for the imaging of live cells: a LSM710 (Zeiss) fitted with a Plan Apo 63x/1.4 oil immersion objective or a TCS SP5 (Leica) fitted with a 63x/1.4–0.6 oil immersion objective. The images are recorded with Zen Black 2012 (Zeiss) or LAS AF 2.4.1 (Leica).
4. Analysis software: All imaging analysis are performed using the MBF version of ImageJ or FIJI^3^ (Schneider et al., 2012).

## Stepwise procedures

### Oocyte preparation

1. The frog is anesthetized in a 2 L beaker containing 50 ml of Tricaine and put in a closed box for 20 min. The nostrils of the animal are left intentionally outside the liquid to prevent drowning during sedation. The leg of the animal is pinched to ascertain complete sedation before dissection.
2. A double pithing is performed. First, hard scissors are placed through the mouth of the frog and the superior part of the skull is cut out. Second, a sharp dissection needle is inserted in the spinal canal to destroy the spinal cord.
3. Two vertical paramedian incisions (2 cm) are made on the left and right side of the abdomen through the skin and the muscles. The ovaries are gently pulled out through the incisions and placed in a 12 cm petri dish containing Ca^2+^-free media to limit cell toxicity during the enzymatic process^4^. Place 5–10 ovarian lobes per dish. If all the oocytes from one frog are taken, 4~6 dishes have to be ready for the collection of the ovaries.
4. The ovarian lobes are then carefully cut open to flatten the sacs and allow access to the enzyme.
5. The tissue is then placed in two 50 ml tubes filled each with 10 ml of the collagenase solution and left on a rocking platform for 1 h. The collagenase solution is then renewed and the cells incubated for another hour.
6. The digestion of the oocytes is carefully monitored by removing a few cells and checking for the efficiency of the digestion under a dissecting microscope. Different batches of collagenase will have different activity, which will affect the timing of the digestion. Over-digestion will decrease oocyte quality. Efficient defolicullation of the oocytes can be assessed by simply staining the cells with the nuclear dye Hoechst 33342 to visualize the nuclei of follicle cells around the oocyte.
7. The cells are then washed 4x with Ca^2+^-free solution and 4x with Ca^2+^-containing Ringer to remove all the collagenase before transferring them to L15 media. Undigested clusters of cells are discarded using a plastic Pasteur pipette and stage VI oocytes can be selected according to their size (above 1 mm). The quality of the oocytes often correlates with the sharpness of the ring separating the animal (dark) and vegetal (yellowish) poles. The oocytes are stored at 18°C and used within a week of their isolation.

### RNA synthesis and microinjection

1. Coding sequences of interest are inserted into the multiple cloning site of the pSGEM vector (Villmann et al., 1997). This vector flanks the multi-cloning site with the 3′ and 5′ untranslated regions of the *Xenopus* β-Globin gene. This helps decrease cRNA degradation once injected in the oocyte.
2. Cloning is confirmed by restriction digestion and sequencing.
3. RNA synthesis is performed according to manufacturer's instructions. The quality of the RNA is always checked on a denaturing formaldehyde agarose gel for size, integrity and concentration.
4. The microinjection needle is first back filled with mineral oil and mounted on the injector. The needle is then slowly tip-filled using a small drop (1–2 μl) of the RNA solution placed onto a piece of parafilm under a stereomicroscope.
5. Oocytes are injected at least 24 h after isolation. The oocytes are held in a plastic grid and bathed into L15 during the injection procedure. The cells are injected in either pole with volumes typically ranging between 9.2 and 46 nl^5^. The amount of RNA to be injected has to be determined for each protein of interest. Typically for electrophysiological recording 5 ng STIM1 and 1 ng Orai1 allows a good signal to noise ratio, the amount can be doubled for confocal imaging purpose.

### Electrophysiological recordings

#### Separating ca^2+^ sources using caCC

One of the main difficulties in monitoring SOCE using electrophysiological techniques is the very small amplitude of the whole cell current (~1 pA/pF). In *Xenopus* oocytes variations in intracellular Ca^2+^ concentrations near the plasma membrane can be monitored using the activation of large CaCC that amplifies the Ca^2+^ signals (Machaca and Hartzell, 1999). The physiological role of these channels is to contribute to the maintenance of the membrane potential of the oocyte close to E_Cl_ and to allow a Ca^2+^ induced depolarization in the egg that blocks polyspermy (Machaca et al., 2001). The currents through CaCC show time and voltage dependent activation kinetics as well as strong outward rectification when the Ca^2+^ concentration below the plasma membrane remains in the sub-micromolar range. The main protein involved in the formation of the channel has been recently identified as a member of the anoctamin family termed TMEM16A or Ano1 (Kuruma and Hartzell, 2000; Machaca et al., 2001; Hartzell et al., 2005; Schroeder et al., 2008; Huang et al., 2012). These channels allow real-time monitoring of the Ca^2+^ concentration below the plasma membrane but give a less adequate image of cytoplasmic Ca^2+^ due to the very efficient recycling of Ca^2+^ in the endoplasmic reticulum (Machaca and Hartzell, 1999). Most important, the difference in voltage sensitivity between CRAC and CaCC and the large difference between a theoretical E_Cl_ of -26 mV in our recording conditions (Costa et al., 1989) and the highly depolarized value of E_Ca_ allows separation between Ca^2+^ release and Ca^2+^ influx in activating the CaCC. This is accomplished using the voltage-clamp protocol described below.

**Figure 1:**
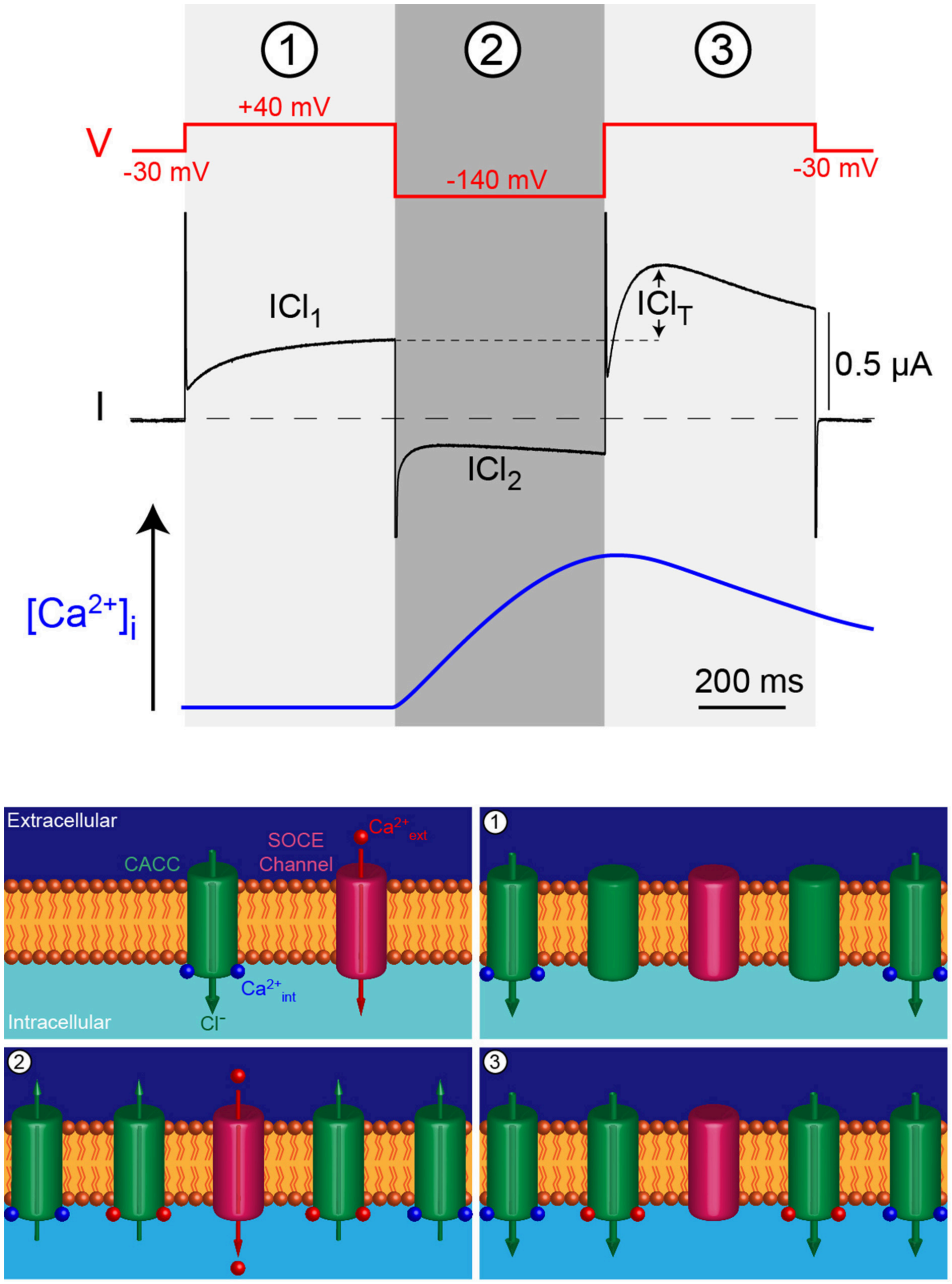
**Triple voltage jump protocol used to monitor the intracellular Ca^**2+**^ concentration ([Ca^**2+**^]_i_) in the ***Xenopus*** oocyte**. The first depolarizing jump (1) measures the Cl^−^ current through CaCC at rest or during Ca^2+^ release from intracellular stores, mirroring [Ca^2+^]_i_. The hyperpolarizing voltage jump (2) drives Ca^2+^ through SOCE channels that in turn induce Cl^-^efflux. The final depolarizing pulse (3) reports the activation of the CaCC by the Ca^2+^ influx produced during the previous hyperpolarizing pulse (blue trace). The difference between current obtained in 1 and 3 is termed ICl_T_ and reports Ca^2+^ influx.

The voltage-clamp protocol is composed of three voltages pulses (Figure 1). Simultaneous sub-membrane Ca^2+^ imaging has been used to monitor the increase in Ca^2+^ concentration induced by the hyperpolarizing voltage pulse to −140 mV, as well as the clearance kinetics after depolarization (Machaca and Hartzell, 1999). The cells are first held at a steady state membrane potential of −30 mV (close to the resting membrane potential), the first pulse to +40 mV then triggers a Cl^−^ influx depending on intracellular Ca^2+^ concentrations ([Ca^2+^]_i_), termed ICl_1_. The second pulse to -140 mV increases the driving force for Ca^2+^ entry through any open Ca^2+^-permeant channel and activates an inward Cl^−^ current, termed ICl_2_ with smaller amplitude than ICl_1_ due to the fast inactivation and outward rectifying properties of the CaCC. The third pulse is identical to the first one (+40 mV, 500 ms), the Cl^−^ current obtained is therefore a reading of [Ca^2+^]_i_ after the second pulse. The difference in amplitude between the Cl^−^ currents obtained in the +40 mV depolarizing pulses 1 and 3 reflects Ca^2+^ influx triggered during the second hyperpolarizing pulse to −140 mV and is termed ICl_T_ (for transient). ICl_T_ decays with similar kinetics as the clearance of intracellular sub-membrane Ca^2+^ (Machaca and Hartzell, 1999).

**Figure 2:**
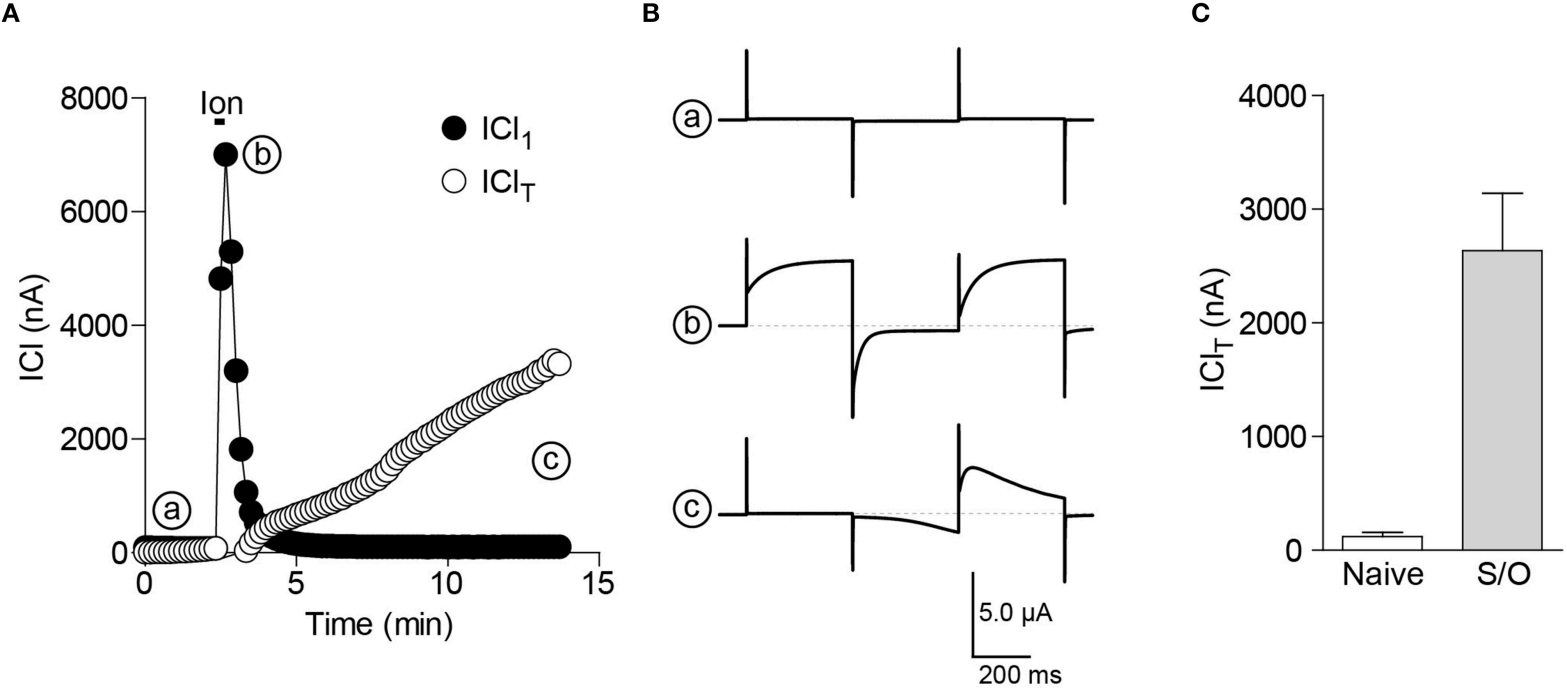
**CaCC as an endogenous sensor for Ca^**2+**^ release and SOCE in the oocyte. (A)** Simultaneous reading of ICl_1_ and ICl_T_ show the transient increase in ICl_1_ (b) during store depletion by ionomycin (10 μM, 20 s) and the following increase in ICl_T_ due to SOCE activation (c). **(B)** Currents traces corresponding to the different time points in **(A)**. **(C)** Bar chart illustrating the increase in the ionomycin-induced SOCE (ICl_T_) in cells expressing STIM1 and Orai1 (S/O) compared to uninjected oocytes (Naïve).

Procedure to measure SOCE using CaCC as readout:

1. The oocytes are injected 2–3 days before experiments with RNA coding for STIM1 (5 ng) and Orai1 (1 ng).
2. Using the voltage clamp protocol described above, the cells are perfused with ionomycin (10 μM, 20 s) to empty the ER stores. The intracellular Ca^2+^ release can be measured as a large transient increase in ICl_1_ (Figures 2A,B).
3. The Ca^2+^ influx induced by SOCE can be monitored as the development of the ICl_T_ component and is larger in STIM1/Orai1 injected oocytes (Figure 2).

#### Measuring SOC current

Although endogenous CaCC are useful tools that amplify and report sub-membrane Ca^2+^ intracellular signals, they do not provide a perfect reporter of SOCE. First, the CaCC do not behave linearly at steady-state across the membrane potential range required to study the SOCE current; second the CaCC are voltage-dependent and third the co-localization or absence of co-localization of CaCC and SOCE proteins might impair the reading of Ca^2+^ influx. It is not possible to obtain sufficient pharmacological inhibition of CaCC in *Xenopus* oocytes, since the amplitude of the chloride currents is 10–100-folds larger than that of SOCE. The solution is therefore to uncouple CaCC from Ca^2+^; this can be achieved using intracellular injection of the fast Ca^2+^ chelator BAPTA (Hartzell, 1996).

**Figure 3:**
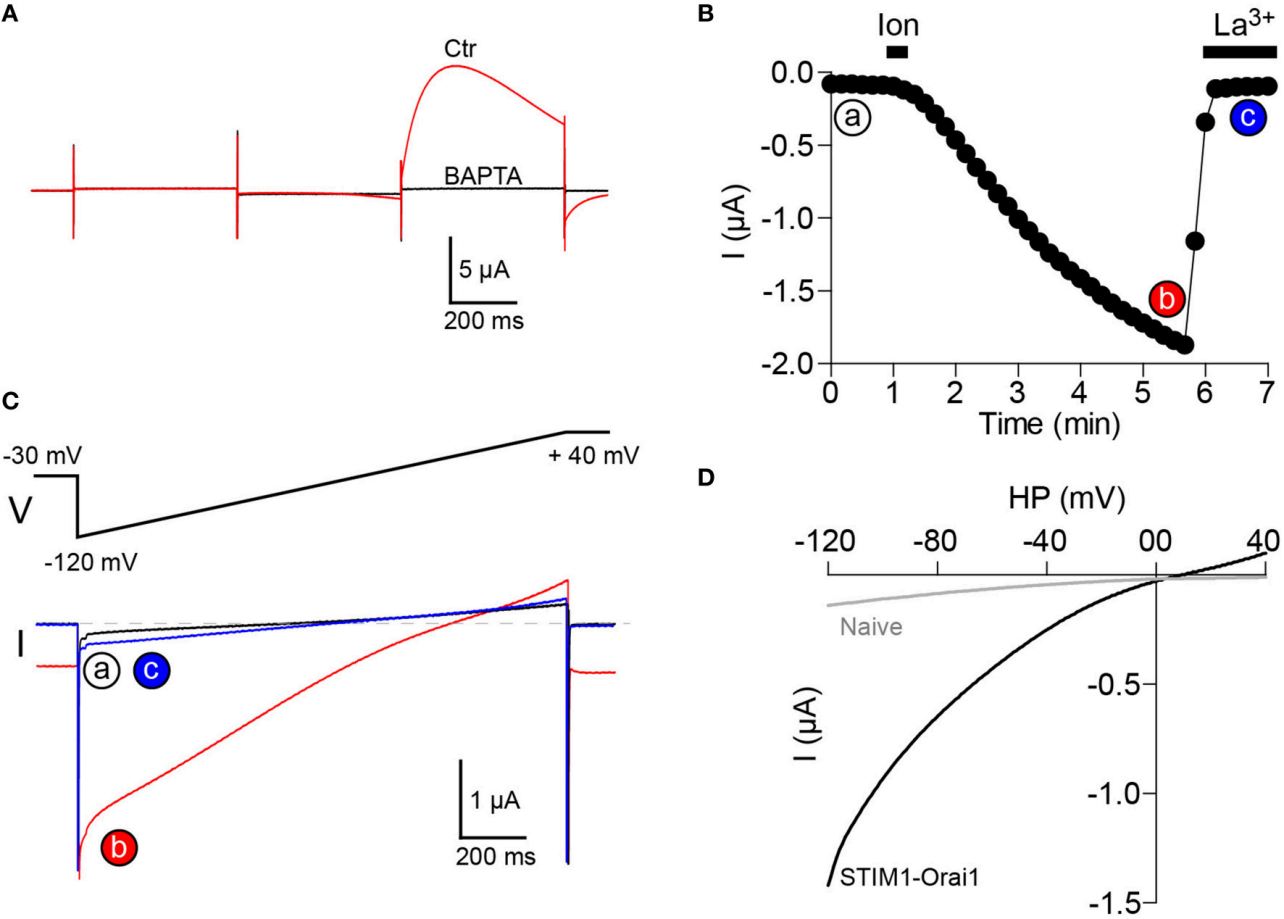
**Recording of SOCE current in ***Xenopus*** oocytes. (A)** Intracellular injection of BAPTA (final concentration 2-5 mM) inhibits CaCC activation. **(B)** The SOCE current is activated by store depletion using bath applied ionomycin (10 μM, 20 s) and is totally inhibited by La^3+^ (100 μM). The current is measured at a membrane potential of −120 mV. **(C)** Current traces obtained using a voltage ramp from −120 mV to +40 mV at time points indicated in **(B)**. **(D)** Current-voltage relationship of the SOCE current (La^3+^-sensitive) obtained after subtraction of the La^3+^ resistant fraction (c) from the global current (b). The current is dramatically increased in oocytes overexpressing STIM1 and Orai1. Recordings in **(A–C)** are made on a cell overexpressing STIM1 and Orai1.

Procedure to measure the SOCE current in *Xenopus* oocytes:

1. A micro-injection pipette (same as the ones used for RNA injection) is tip-filled with a stock solution of 250 mM BAPTA and inserted into the oocyte. The required amount of BAPTA to be injected will depend on the depth of the pipette tip in the oocyte and on the size of the Ca^2+^ influx, typically injecting 10–20 nl of BAPTA is enough to block CaCC activation^6^. This gives a final concentration of intracellular BAPTA ranging from 2.5 to 5 mM. The injection of BAPTA can be performed after store depletion and recording of the ICl_T_ current (Figure 3A), or at the beginning of the experiment before activation of SOCE as illustrated in Figure 3B.
2. To increase the amplitude of the recorded current, the extracellular Ca^2+^ can also be raised from 1.8 to 5 mM.
3. The SOCE current is studied using a voltage ramp from −120 mV to +40 mV.
4. To measure the actual size, time course and voltage dependence of the SOCE current, La^3+^ (100 μM) is bath applied to fully inhibit the SOCE current (Figures 3B,C).
5. The remaining La^3+^-resistant current can then be subtracted from the current trace obtained after store depletion. This results in a La^3+^-sensitive current that displays the typical inward rectification of SOCE currents and is dramatically potentiated following STIM1 and Orai1 co-expression in the oocyte (Figure 3D). For a more specific inhibition of SOCE currents, the inhibitor BTP-2 (10 μM) can be used (Courjaret and Machaca, 2014). We do not recommend the use of 2-APB, although it also inhibits the SOCE current in oocytes, 2-APB clearly has multiple targets and is not a selective inhibitor (unpublished results).

### Confocal imaging

#### Visualizing STIM1 and orai1 clustering

An important advantage of the *Xenopus* oocyte as a model system to study SOCE is its large size, which allows good separation and visualization of the subcellular distribution of STIM1 and Orai1 as well as SERCA, the main players mediating SOCE (Courjaret and Machaca, 2014).

**Figure 4:**
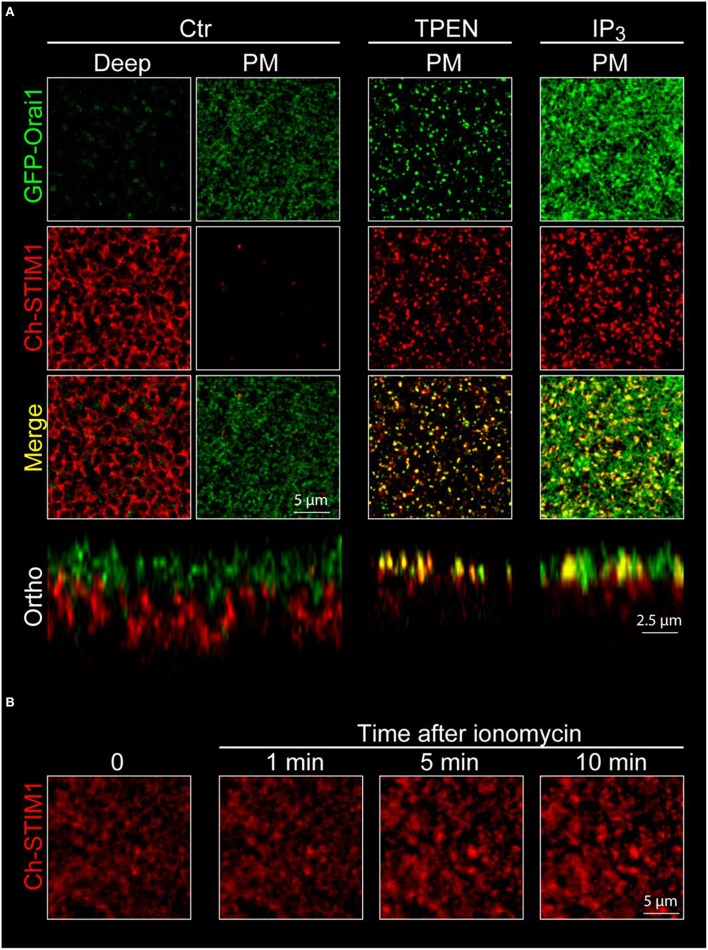
**Confocal imaging of STIM1 and Orai1 in X***enopus*** oocytes. (A)** Confocal z-stacks are obtained from oocytes overexpressing mCherry tagged STIM1 (Ch-STIM1) and GFP-Orai1. The pinhole aperture is set to 1 Airy unit and the images are taken every 0.5 μm. Two selected planes are illustrated, the Plasma Membrane (defined as the maximum expression level of GFP-Orai1) and the intracellular/ER compartment (Deep) approximately 10 μm deep in the cell and at the maximum of Ch-STIM1 expression in control conditions (Ctr). Treatment of the cells with TPEN (5 mM, 10 min), IP_3_ (2.5 μM final) induces the translocation of Ch-STIM1 to the plasma membrane and the co-clustering of the proteins, visible as dense yellow dots in the merged images. Below each panel (Ctr and TPEN) is an orthogonal reconstitution (Ortho) of a plane perpendicular to the membrane illustrating the distribution of both proteins across the z-axis. **(B)** Timeline of the formation of Ch-STIM1 clusters following application of ionomycin (10 μM). The images are captures using an open pinhole aperture on the confocal to compensate for the vertical movement of STIM1.

**Figure 5:**
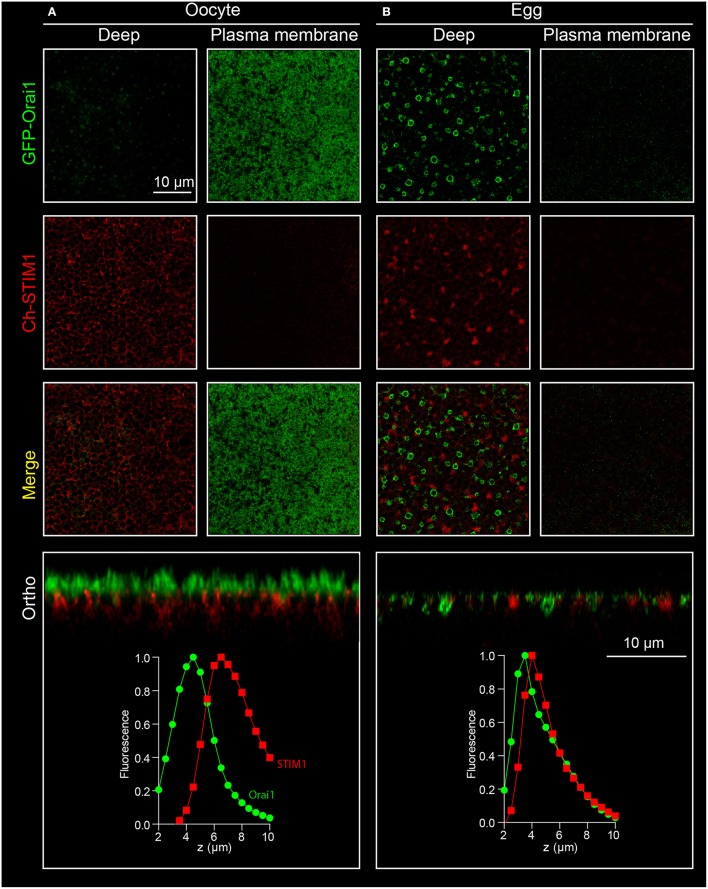
**Orai1 internalization during meiosis**. Confocal stacks are performed on oocytes **(A)** and eggs **(B)** expressing both STIM1-Ch and Orai1-GFP. Oocyte maturation is associated with internalization of Orai1. This is visible as the removal of Orai1 from the plasma membrane in the oocyte and its enrichment in an endosomal compartment in confocal sections (deep), on the orthogonal reconstruction (ortho) and on the fluorescence curves as a shift of the Orai1 intensity curve toward the ER as marked by STIM1.

Both STIM1 and Orai1 proteins are tagged at their N-terminal end using mCherry and GFP respectively. RNAs coding for mCherry-STIM1 (10 ng) and GFP-Orai1 (2 ng) are injected into the oocyte and allowed 2–3 days to express (Yu et al., 2009) after which confocal imaging is performed as described below:

1. The recording chamber is filled with Ringer and the oocyte is dropped in the metal holder. At this point the decision is made to image either the animal or vegetal pole of the cell^7^.
2. Although the cell is very thick, with maximum trans-illumination the black pigmentation of the membrane allows to focus the microscope lens on the desired area before starting laser scanning.
3. The confocal pinhole is set to 1 Airy unit. Z-stacks are performed across the plasma membrane by steps ranging from 0.25 to 1 μm depending on the precision needed. Typically scans are done on a 1024 × 1024 pixel frame using an averaging of 2 lines per channel and scanning up to 20 μm deep in the cell.
4. The first signal detected is the tips of the microvilli of the oocytes appearing as bright Orai-GFP-positive dots in the confocal optical slice (Figure 4, plasma membrane, green channel). As the scan goes deeper in the cell the Orai1-GFP signal goes down and the Ch-STIM1 signal appears. The reticular pattern seen in Figure 4A (deep, red channel) is typical of an intracellular/ER expression of the protein that is excluded from the spherical pigments granules that appear as dark circles on the images.
5. Orthogonal sectioning can then be performed either offline using image analysis software or live if the instrument allows “xz” scans. As can be seen in the lower left panel of Figure 4 the spatial separation between the ER and the plasma membrane is clear.
6. The cells are incubated with TPEN (5 mM, 10 min in Ringer) to mimic store depletion^8^, and promote the formation of the STIM1-Orai1 clusters at the membrane. To speed up the imaging process, cells are first imaged in control conditions, then incubated with TPEN and imaged again. This allows the recording of multiple cells in a shorter time (a cell can be imaged during the incubation of another one). Similar results can be obtained using the injection of IP_3_ or its non-hydrolyzable analog IP_3_df (to a final concentration of 2.5 μM and 100 nM respectively) or by depleting the stores with a short term (20 s) application of ionomycin (10 μM) (Figures 5A,B).
7. The same z-stacks reveal large bright clusters at the plasma membrane level in both channels indicating the translocation of STIM1 to the cortical ER, as well as the co-clustering of the two proteins. The cortical ER following store depletion localizes to the same focal plane as the plasma membrane.

#### Visualizing orai trafficking

Besides the visualization of protein translocation and clustering during store depletion, the movement of membrane proteins between intracellular compartments and the plasma membrane can also be studied in the *Xenopus* oocyte (Yu et al., 2009, 2010). A critical change in Orai1 distribution can be observed during oocyte meiosis, where Orai1 is removed from the cell membrane and internalized into a late endosomal compartment (positive for Caveolin, Rab5, Rab7, and Rab9) resulting in a complete loss of the SOCE current in the egg (Yu et al., 2009, 2010).

Procedure to visualize Orai1 internalization:

1. The oocytes are injected 2–3 days prior to step 2 with 1–2 ng of RNA coding for GFP-Orai1. This can be done with or without co-expression of other proteins of interest. In the example shown in Figure 5 Orai1 is co-expressed with STIM1.
2. The oocytes are matured to eggs by incubating them overnight in L15 medium containing progesterone (5 μg/ml).
3. Eggs can be spotted by the white spot in the animal pole indicating Germinal Vesicle Breakdown (GVBD). This can be further confirmed by fixing the oocytes and cutting them in half to visualize the breakdown of the nuclear envelope.
4. Confocal imaging is performed on oocytes and eggs according to the procedure described above in Section Visualizing STIM1 and Orai1 clustering.
5. On the egg, Orai1-GFP fluorescence is reduced at the plasma membrane and is enriched in intracellular vesicles (Figure 5).

## Anticipated results and data analysis

### Electrophysiology

After store depletion, the ICl_T_ current development is visible in cells overexpressing STIM/Orai as well as in naïve oocytes. As stated before, the size of the ICl_T_ is measured using the peak difference between the current induced by the voltage pulse to +40 mV before and after the −140 mV hyperpolarizing pulse. The increase in the current amplitude when overexpressing SOCE proteins is very important (Figure 2C). If too much cRNA is injected (i.e., more than 10 ng of STIM and 2 ng of Orai per oocyte) or if the duration of expression exceeds 72 h the resulting ICl_*T*_ current might be too large to be correctly clamped. Variability of expression levels will exists between oocytes and between frogs. To minimize it, it is better to inject a mixture of both STIM and Orai cRNAs than injecting twice. For simple recordings (to determine current vs. no current) a small number of cells (5–10) will give an adequate reading. When expressing STIM or Orai mutants, a larger number of cells (and frogs) will be required to detect any effect on the ionic currents. Statistical analysis is usually performed using an unpaired Student “t” test and if more than two conditions are used an ANOVA test is used. There is usually no need to normalize the current amplitude unless some great variability appears in the current obtained with the wild type proteins. The currents in the naïve cells being very small when compared to overexpressing ones it is not advisable to use them as a normalization reference.

### Confocal imaging

#### Quantifying STIM1 translocation and clustering

Several parameters can be extracted from the confocal images to define the behavior of STIM1 and Orai1 during store depletion (Courjaret and Machaca, 2014).

**Figure 6:**
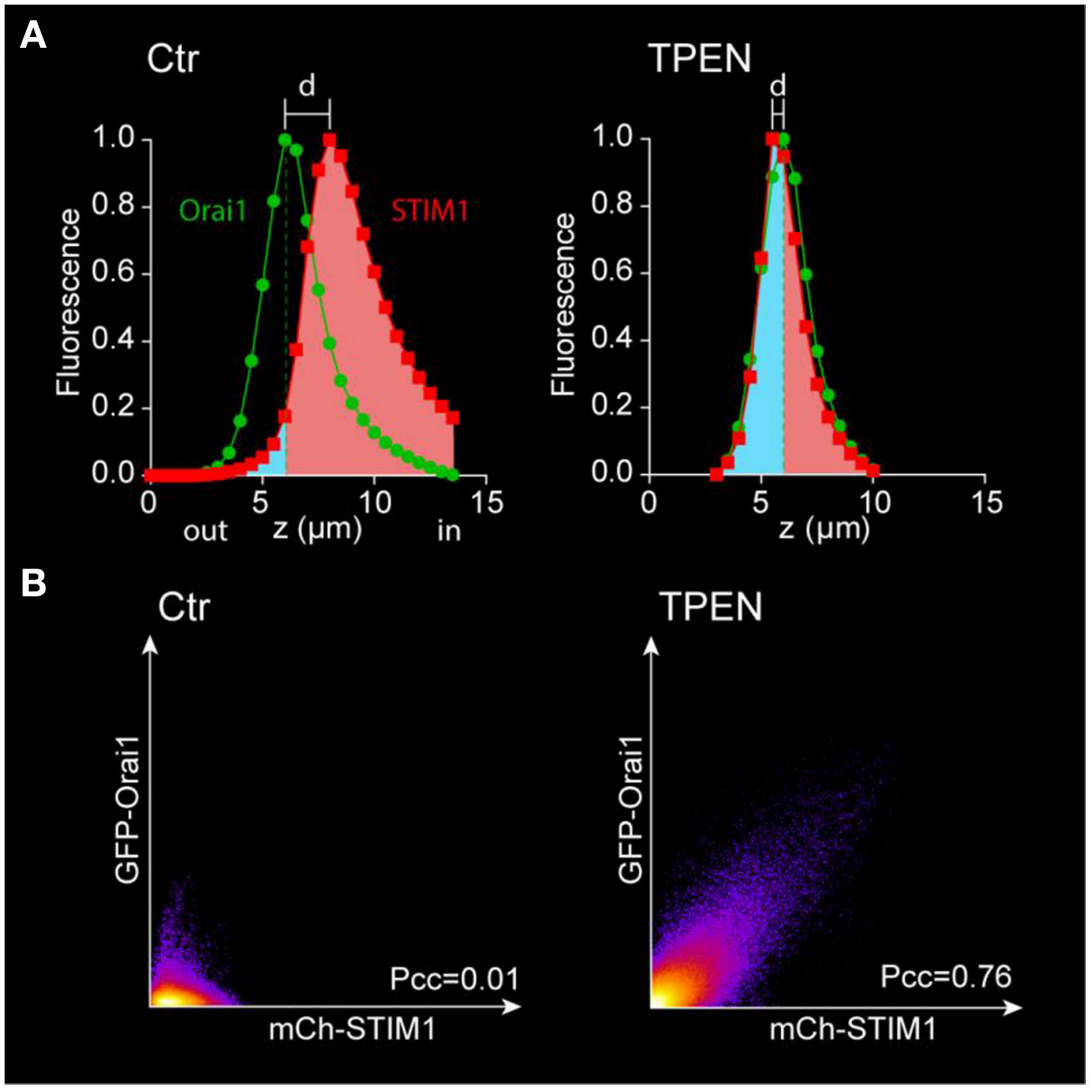
**Analysis of the movement of STIM1 and Orai1 after store depletion. (A)** The fluorescence intensity of each channel is plotted vs. the position on the z-axis and normalized to its maximum. The separation between the Ch-STIM1 and GFP-Orai1 peaks disappears after store depletion induced by TPEN due to co-localization of both proteins at the membrane focal plane as illustrated in Figure 4. The translocation of the STIM1 proteins can also be highlighted using the area under the curve with a reference point set at the peak of the Orai1 signal. The intracellular fraction is labeled in pink and the “membrane” one (above the Orai1 peak) in blue. **(B)** Co-localization plots of mCh-STIM1 and GFP-Orai1 fluorescence obtained at the membrane plane (defined as the maximum of the GFP-Orai1 signal) before and after store depletion by TPEN. The Pearson's correlation coefficient (Pcc) is indicated on each plot.

1. The fluorescence intensity of each channel of each optical slice is normalized to the maximum value of the stack and is plotted as a function of its position on the z axis (Figure 6A)^9^. The distance between the two peaks (*d*) reflects the distance between the Ch-STIM1 and GFP-Orai1 protein populations.
2. After store depletion with TPEN, the translocation of Ch-STIM1 to the cortical ER can be measured as a dramatic reduction in *d*.
3. An alternative method is to define the peak of the Orai1 curve as a reference for plasma membrane plane and to measure the area under the curve of the STIM1 signal at the left and right of the Orai1 maximum (Figure 6A). This gives a good indication of the movement of proteins toward the plasma membrane and improves the signal to noise allowing more accurate statistical analysis.
4. The enhanced co-localization of Ch-STIM1 and GFP-Orai1 in the clusters can be visualized in the co-localization plots (Figure 6B), and measured as an increase in the Pearson's correlation coefficient (Pcc^10^).

#### Measuring clusters

Cluster numbers, area and distribution can be measured using the ImageJ “Analyze particles” plugin (Courjaret and Machaca, 2014).

**Figure 7:**
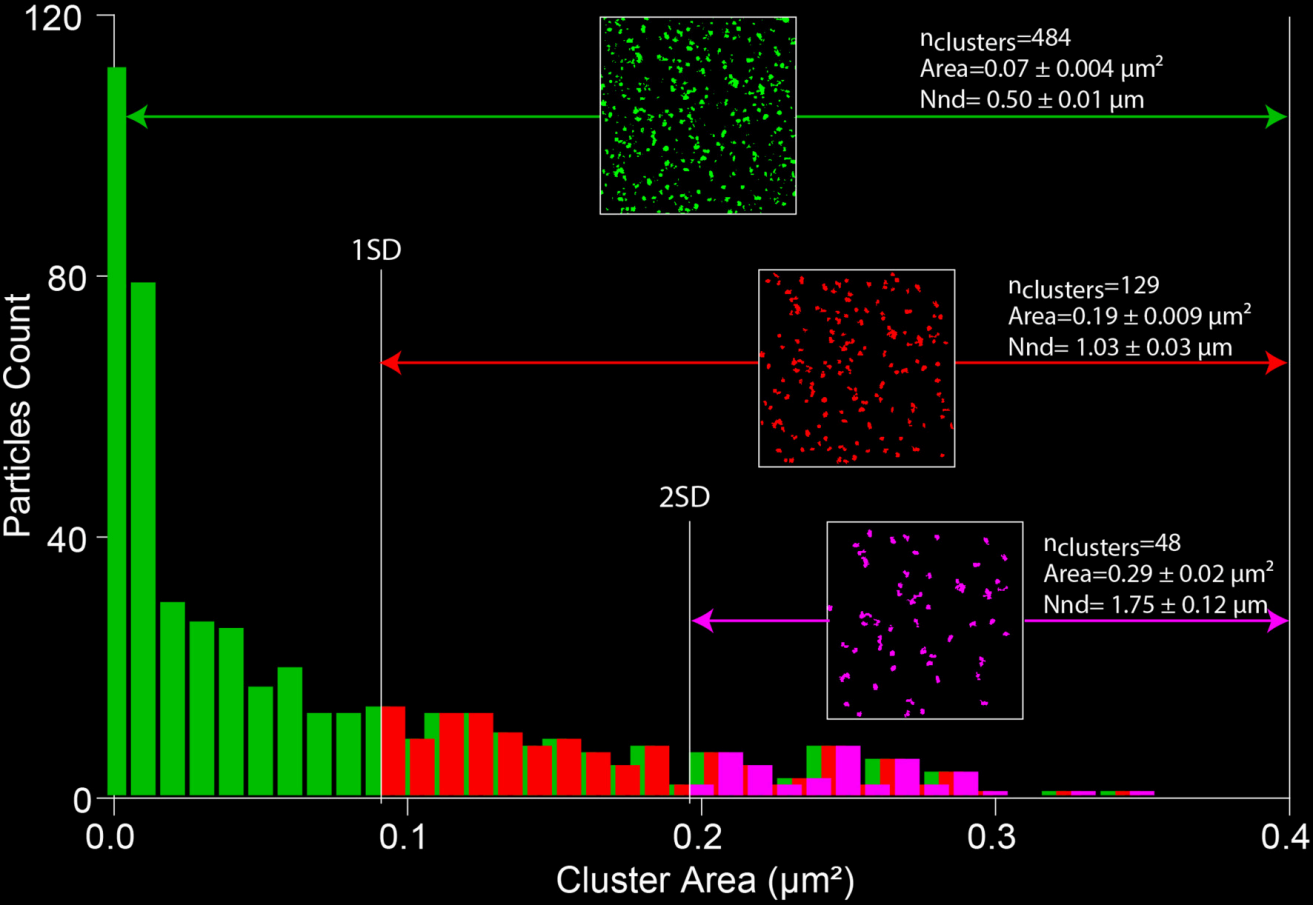
**Measuring SOCE clustering parameters**. A thresholded image of the Orai1 clusters (from Figure 4) is processed with the “analyze particle” plugin of ImageJ. The distribution of the areas of individual clusters is analyzed and the SD of the data is used to define a cutoff value that allows limiting the size of the analyzed particles. The nearest neighbor distance gives a measure of the cluster density (NND).

1. The selected image has to be thresholded first to remove most of the non-specific low intensity pixels and to create a binary image (Figure 7).
2. Using the “Analyze particles” plugin, the size is set from 0 to infinity, therefore detecting all cluster sizes. This parameter can be adapted depending on the noise of the images.
3. The area of each individual clusters is analyzed to determine the standard deviation. The small particles can be excluded from the analysis using thresholds based on the SD value. Figure 7 illustrates the change in size, number and distribution of the detected clusters depending on the defined threshold.
4. Two parameters are then extracted from the data: the surface of each individual cluster (area) and the cluster density. The density is evaluated by measuring the distance between the centers of each cluster using the “Nearest Neighbor Distances” plugin of image J^11^. Those values are of key importance when evaluating the size of the Ca^2+^ microdomain and the localization of proteins in or away from the cluster (Courjaret and Machaca, 2014).

#### Measuring orai trafficking

To measure Orai internalization in the egg, since Orai is moving toward the cytoplasm, it cannot be used anymore as a membrane reference. Then either the STIM1 maximum is used as an intracellular reference (although the ER remodels in the egg) or another reference has to be chosen at the plasma membrane (Figure 5). The CaCC Ano1 can be overexpressed and tagged with mCherry and does not internalize in the egg and can therefore serve as a membrane reference (Yu et al., 2010). Another option is to label the cell membrane with Wheat Germ Agglutinin taking care to keep the cells cool (4°C) to reduce internalization of the plasma membrane (El Jouni et al., 2008).

## Conclusions

In terms of Ca^2+^ signaling the oocyte expresses a very small number of Ca^2+^-permeable channels at the plasma membrane (probably resticted to Orai1 and TRPV6) and the Ca^2+^ influx at rest is reduced. It therefore provides a very good background for recording overexpressed channels. Similarly, the only source for intracellular Ca^2+^ release from the ER is the IP3 receptors creating as simple mechanism of store depletion. The size of the oocyte allows the simultaneous recording of the formation of the SOCE clusters in 3 days (meaning the translocation of the ER toward the plasma membrane can be visualized and no only cluster formation in one plane) as well as the development of the Ca^2+^ current using CaCC as amplifiers.

Using the different experimental approaches described in this chapter, in the frog oocyte one can study structure-function properties of STIM1 and Orai1 (Yu et al., 2011), the basic regulation of SOCE during the cell cycle (Machaca and Haun, 2000, 2002; Yu et al., 2009), Orai1 trafficking (Yu et al., 2009) and the contribution of SOCE to global and precise Ca^2+^ signaling (Courjaret and Machaca, 2014). The possibility to inject modified version of the SOCE proteins in single oocytes makes it a very powerful model system to better understand SOCE physiology and regulation at the single cell level.

## Author contributions

RC performed experiments and analyzed data. KM analyzed data. RC and KM developed methods and wrote the manuscript.

### Conflict of interest statement

The authors declare that the research was conducted in the absence of any commercial or financial relationships that could be construed as a potential conflict of interest.

## Footnotes

^1^Wild animals are preferred to lab bred ones and provide more oocytes and less variability from one batch to another. Animals were handled according to Weill Cornell Medicine College IACUC approved procedures (protocol #2011-0035).

^2^When possible select the injection needles that have a beveled tip after breaking it with the tweezers. A blunt needle does not penetrate the oocytes as good.

^3^The version of Image J used is the distribution created by the Mac Master Biophotonics Facility (Ontario, Canada, http://imagej.net/mbf/). An alternative is to use the FIJI distribution (http://fiji.sc/Fiji). The different plugins can be found on the ImageJ website (http://rsb.info.nih.gov/ij/index.html). To correctly import files created for different microscopes it is critical that the “LOCI Bio Format Library” is up to date.

^4^A window can also be open in the abdomen of the frog to gain a better access to the oocytes and to reduced accidental contact to the skin of the frog and prevent contamination.

^5^Although injector can inject volumes smaller than 9.2 nl, the risk of having failed injections is increased. Large injected volumes (>50 nl) might affect the cell and sham injection are required to monitor any stress-induced effect.

^6^The effect of BAPTA occurs within minutes of injection. Insufficient block of the CaCC is visible on I/V curves when a significant outward current is present for values more depolarized than 0 mV.

^7^The vegetal pole of the oocyte being less pigmented than the animal pole, it is often preferable to image that hemisphere to reduce background signal. However, the fact that regionalization of the expression can occur has to be taken into account and for any new protein expressed, both sides have to be observed in the preliminary experiments.

^8^Several agents can be used to promote cluster formation and SOCE including actual stimulation of IP_3_ receptors (Figure 4). Ionomycin is preferred during electrophysiological experiments since it induces robust currents and allows real time monitoring of the SOCE current development and of the emptying of the stores. TPEN is sometimes preferred for imaging experiments since it promotes the formation of clear and stable clusters. TPEN in fact simulates store depletion without allowing Ca^2+^ to be released from intracellular stores. This is because TPEN is a low affinity Ca^2+^ chelator that is freely membrane permeant. TPEN binds the high free Ca^2+^ in the lumen of the ER and as such simulates store depletion without Ca^2+^ leaving the ER because the TPEN-Ca^2+^ complex is charged and becomes membrane impermeant. As such TPEN is able to chelate free ER Ca^2+^. Once free Ca^2+^ levels drop STIM1 loses its bound Ca^2+^ and clusters in a similar fashion to after Ca^2+^ release from the ER in response to agonist stimulation and the activation of the IP3R. This has now been clarified in the text.

^9^The experiments shown are meant to be representative for the purposes of illustrating the methodology described. The statistical approaches and replications for each approach are described in more details in the cited papers. The example of STIM1 and Orai1 overexpression illustrates this point. For these experiments oocytes were injected with STIM1 and or Orai1 from the same donor female within the week of performing the experiment. Uninjected oocytes represent the background fluorescence, i.e., no fluorescence signal. Oocytes injected with the RNA encoding STIM1 and/or Orai1 were imaged under the same laser intensity and PMT gain settings where no signal is apparent on control oocytes. Once those settings are determined for the experiment for the week in question and from the specific donor female there are maintained for all imaged oocytes allowing comparison between the expression levels between different oocytes. This is important because some oocytes don't express and some express very high levels of the protein encoded by the inject RNA. A minimum of 10 oocytes are imaged for any particular setting. For each condition there will be a control expression condition to compare to the experimental conditions.

^10^A limitation to the co-localization analysis is the fact that the STIM and Orai1 do not share a maximum on the same focal plane before store depletion. If absolute values are needed to compare cluster formation a third “non-clustering” membrane protein can be expressed in parallel. An alternative is to perform the co-localization analysis on orthogonal sections and not on XY planes.

^11^The “NND” plugin only works if the “Centroid” measure has been activated in the measuring window of ImageJ.
